# Conformance-Based Doping Detection for Cyber-Physical Systems

**DOI:** 10.1007/978-3-030-50086-3_4

**Published:** 2020-05-13

**Authors:** Rayna Dimitrova, Maciej Gazda, Mohammad Reza Mousavi, Sebastian Biewer, Holger Hermanns

**Affiliations:** 8 IMDEA Software Institute, Pozuelo de Alarcón, Spain; 9 University of Salzburg, Salzburg, Austria; 10 Department of Computer Science, University of Sheffield, Sheffield, UK; 11 School of Informatics, University of Leicester, Leicester, UK; 12 Saarland University - Computer Science, Saarland Informatics Campus, Saarbrücken, Germany

## Abstract

We present a novel and generalised notion of doping cleanness for cyber-physical systems that allows for perturbing the inputs and observing the perturbed outputs both in the time– and value–domains. We instantiate our definition using existing notions of conformance for cyber-physical systems. We show that our generalised definitions are essential in a data-driven method for doping detection and apply our definitions to a case study concerning diesel emission tests.

## Introduction

System doping, in our terminology, is an intentional intervention causing a change in the system’s normal behaviour against the interests of the user or other stakeholders (such as the society at large). Examples of system doping are widespread and range from vendors’ enforcing a monopoly on chargers and spare parts (by checking for and refusing third-party chargers and spare parts, respectively) to tampering with exhaust emission in order to detect and pass emission tests. Doping can be the result of embedding a piece of code or smuggling a piece of electronic circuit into the system and it can be caused by the original developers or by hackers. Software and system doping has been studied in the past couple of years and rigorous theories for it have been developed [8, 9, 15]. These theories were subsequently adopted in order to detect doping, or formally, to check system cleanness [10, 32] (corresponding to the absence of doping).

In the present paper, we extend the theory of doping to the setting of cyber-physical systems (CPS) by exploiting the notions of conformance testing for CPS [1, 17, 33]. The existing theories of software doping define doping in terms of drastic deviations in output as a result of minor deviations in input, where the term “deviation” refers to differences in validity of propositions or values of variables. However, the current notions come short of properly dealing with the issues of retiming and delays, which are commonly present in the signals of CPS. We observe that this is an essential aspect of detecting doping for cyber-physical systems: often the traces to be tested for doping have subtly different timing behaviour, e.g., due to measurement and calibration errors or due to the slight deviations of human actors in acting upon the planned scenarios. The insufficient treatment of retiming and delays can both lead to false negatives, i.e., missing cases of doping, as well as false positives, i.e., reporting spurious doping cases.

To address these issues, we exploit the notion of conformance to devise a general theory of being clean from doping and instantiate that theory with some existing notions of conformance for hybrid systems. We show how these notions can account for retiming and lead to more precise notions of cleanness.

We illustrate the usefulness of our theory by empirical analysis of diesel engine exhaust emissions in the context of one of the official test cycles, the New European Driving Cycle (NEDC) [42]. In particular, we show that catering for retiming is essential in effectively exploiting the actual driving cycles for performing doping analysis. We thus demonstrate that our new theory remedies a major shortcoming in the existing notions from the literature. To facilitate the presentation, we use throughout the remainder of this paper the following simple running example, which is inspired by our case study.

**Fig. 1.**
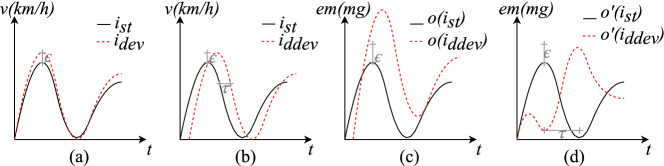
Running example: specified (a) and actual (b) test cycles and emission footprints obtained from different (fictitious) vehicles (c) and (d).

### Example 1

Figure 1.(a) shows two test cycles (evolution of speed over time), designed to detect whether the exhaust emission control of a particular vehicle is doped. The test cycle $i_{ st }$, depicted with a black solid line, is the standard one prescribed by the (fictitious) official regulation, while test cycle $i_{ dev }$, depicted by a red dotted line, is a slight deviation thereof. If the exhaust emissions measured during the test cycle $i_{ dev }$ turn out to be significantly higher than the ones measured in test cycle $i_{ st }$, then we can conclude that the exhaust emission system is potentially doped, since it appears tailored to the standard test cycle.

Figure 1.(b) addresses a notorious problem of testing cars: a human tester is supposed to drive the car as just described, however, she can do this only up to a certain imprecision. Assume her driving of $i_{ dev }$ exhibits a slight time shift $\tau$ relative to the test cycle, as in $i_{ ddev }$, while $i_{ st }$ is being driven as intended.

The result of a test is the emission footprint measured at the exhaust pipe of the car. Figure 1.(c) and Fig. 1.(d) show two different possible test results (obtained from different cars) for the scenario in Fig. 1.(b). Intuitively, the footprints in Fig. 1.(c) provide significant evidence for doping – a slightly different test cycle has resulted in significantly larger footprint. However, due to the time shift on the input side Fig. 1.(b) the point-wise difference of the two driven test-cycles has grown very large. As we show in the remainder of this paper, the existing theory of doping fails to detect such a clear evidence, due to the minor delay during the execution of the driving cycle. The emission footprint in Fig. 1.(d) is another (synthetic) example of a significant deviation which cannot be detected for the input in Fig. 1.(b) using existing theories; this latter footprint sheds some light on the intricate design decisions in the theory we develop in this paper.

The contributions for this paper can be summarized as follows:

- We define a *general notion of conformance* that can express different ways of comparing execution traces by allowing deviations both in value and in time.
- We define a general *notion of cleanness for hybrid systems*, and show that it subsumes the existing notion of robust cleanness [15].
- We demonstrate the usefulness of the proposed generic framework by applying it to *software doping tests* in the automotive domain, where we show that the new cleanness definition is able to flag a case of software doping that goes unnoticed when robust cleanness is used.

## Related Work

The term “software doping” was coined around 2015 [30] in media uncovering the diesel exhaust emissions scandal. An informal problem formulation [8] pointed out the general phenomenon of intentionally added hidden software behaviour, which is not in the interest of the consumer. Shortly after, this observation has been complemented by a set of formal *cleanness* definitions [15] laying the theoretical foundations upon which formal methods to detect such software behaviour can be used. It is possible to detect missing functionality and undesired existing functionality. The definitions support both sequential programs and non-deterministic reactive programs. To check satisfaction of the definitions, it is necessary to compare two (or more) execution traces of the same system. Such properties are called *hyperproperties* [13] (whereas classical properties are *trace properties*). Tool support for analysing hyperproperties typically requires high computational effort [12, 25]. There exist several temporal logics for analysing satisfaction of trace properties of various kinds of systems, one of them being *Linear Temporal Logic* (LTL) [39] for systems producing outputs in discrete time steps and properties that do not consider the time passing between outputs. LTL has been extended to the logic HyperLTL, which can express hyperproperties by allowing explicit quantification of execution traces in front of an LTL formula [12]. Tools for model-checking boolean circuits, satisfiability and monitoring of HyperLTL specifications have been developed [6, 11, 21–25, 29].

*Signal Temporal Logic* (STL) [36] is an extension of LTL that adds support for time constraints and real-valued signals. Tools exist that automatically try to falsify STL formulas [7, 18]. There has been an extension of STL to HyperSTL in a similar fashion as it was done for HyperLTL [37]. The syntax of HyperSTL, however, is not able to express the cleanness definitions (for deterministic systems) in a way that allows (efficient) falsification. *Robust cleanness* is defined for distance functions on inputs and outputs [15]. When used with temporal logics the distance functions are restricted to those compatible with the logics. To be fully independent, robust cleanness analysis has been embedded into the theory of model-based testing [10] with input-output conformance [40, 41].

Notions of conformance for discrete event systems have been discussed for almost a century. The earliest work on this topic dates back to 1960’s when researchers studied model-based testing of digital circuits using Finite State Machine models [31, 35]. Concurrency theory contributed ideas to this field, such as decoupling (i.e., removing the synchronised assumption between) inputs and outputs and observing failures to engage in a communication (and more specifically quiescence) [16, 40]. A theory of conformance testing for systems with continuous dynamics was developed by Michiel van Osch [38]; this theory did not gain much popularity in practice, partly because of its insufficient treatment of approximation (e.g., differences in values and retiming). Pappas and Girard [27, 28] proposed the use of Metric Bisimulation for conformance checking in dynamical systems and Pappas and Fainekos [20] developed a falsification framework for the same purpose. This research led to two notions of conformance used in the present paper, namely hybrid conformance by Abbas and Fainekos [1] and Skorokhod conformance by Deshmukh, Majumdar, and Prabhu [17].

## Preliminaries

*Semantic Domain.* In this section, we provide definitions regarding semantic domain, conformance, and robust cleanness. We begin with the definition of our semantic domain, called generalised timed traces [26]. This definition subsumes both discrete-time state sequences and continuous-time trajectories. A generalised timed trace is a function with a discrete or continuous domain (called time domain) and a co-domain which is a metric space. Intuitively, a generalized timed trace maps each element of its time domain to a state. We require that the set of possible states is a metric space since we study conformance notions that compare traces based on the distance between the states of the traces.

### Definition 1

Let $(\mathcal{Y},d_\mathcal{Y})$ be a metric space. A ${\mathcal{Y}}$-valued *generalised timed trace (GTT)* is a function $\mu : \mathcal{T}\rightarrow \mathcal{Y}$ such that $\mathcal{T}\subseteq \mathbb {R}_{\ge 0}$. We call $\mathcal{T}$ the *time domain* of $\mu$, denoted $dom (\mu )$. $GTT (\mathcal{Y})$ is the set of all ${\mathcal{Y}}$-valued generalised timed traces.

For a GTT $\mu :\mathcal{T}\rightarrow \mathcal{Y}$ and time $t_0 \in \mathcal{T}$, by $\mu [\dots t_0]$ we denote the prefix of $\mu$ up to $t_0$, i.e., the restriction $\mu |_{t \in \mathcal{T}: t\le t_0}$; likewise, by $\mu [t_s \dots t_e]$, we shall denote the restriction $\mu |_{t \in \mathcal {T} : t_s \le t \le t_e}$

A hybrid system is a mapping from generalised (input) traces to sets of generalised (output) timed traces.

### Definition 2

A ${\mathcal{Y}}$-valued hybrid system is a function $H : GTT (\mathcal{Y}) \rightarrow$ $\mathcal{P}( GTT (\mathcal{Y}))$ such that for all $\mu \in GTT (\mathcal{Y})$ and all $\mu ' \in H (\mu )$ it holds that $dom (\mu ') = dom ( \mu )$. We define $\mathcal {H}(\mathcal{Y})$ to be the set of all ${\mathcal{Y}}$-valued hybrid systems.

In addition, we distinguish deterministic hybrid systems whose output values range over singleton sets only. In what follows, we identify deterministic hybrid systems with functions of the type $GTT (\mathcal{Y}) \rightarrow GTT (\mathcal{Y})$.

For simplicity, we assume that the input and output domain are defined on the same metric spaces. The generalisation to different spaces is straightforward.

*Conformance Relations.* Recently, a number of notions of conformance for cyber-physical systems have been proposed [3, 33]. It turns out that these notions, two of which are quoted below, can provide a rigorous basis for doping detection.

Note that throughout the paper, the variables $\tau$ and $\epsilon$ (with possible subscripts) always range over non-negative real numbers.

### Definition 3

We say that $\mathcal{Y}$-valued GTTs $\mu _1:\mathcal{T}_1 \rightarrow \mathcal{Y}$ and $\mu _2:\mathcal{T}_2 \rightarrow \mathcal{Y}$ are:

- *trace conformant* with tolerance threshold for signal value $\epsilon$, notation $\textsf {TraceConf}_{\epsilon }(\mu _1,\mu _2)$, if $\mathcal{T}_1=\mathcal{T}_2$ and for all $t \in \mathcal{T}_1$, $d_{\mathcal{Y}}(\mu _1(t),\mu _2(t)) \le \epsilon$
- *hybrid conformant* with thresholds $\tau$ and $\epsilon$, denoted $\textsf {HybridConf}_{\tau ,\epsilon }(\mu _1,\mu _2)$, if:
  - $$\forall t_1 \in \mathcal{T}_1 \,\exists t_2 \in \mathcal{T}_2:\, |t_2-t_1|\le \tau \,\wedge \, d_{\mathcal{Y}}(\mu _2(t_2),\mu _1(t_1))\le \epsilon $$
  - $$\forall t_2 \in \mathcal{T}_2 \,\exists t_1 \in \mathcal{T}_1:\, |t_1-t_2|\le \tau \,\wedge \, d_{\mathcal{Y}}(\mu _1(t_1),\mu _2(t_2))\le \epsilon $$
- *Skorokhod conformant* with tolerance thresholds $\tau$ and $\epsilon$, notation $\textsf {SkorConf}_{\tau ,\epsilon }(\mu _1,\mu _2)$, if $\mathcal{T}_1$ and $\mathcal{T}_2$ are intervals and there is a strictly increasing continuous bijection $r:\mathcal{T}_1 \rightarrow \mathcal{T}_2$ called retiming, such that:
  - for all $t\in \mathcal{T}_1$, $|r(t)-t| \le \tau$, and
  - for all $$t\in \mathcal{T}_1$$, $$d_{\mathcal{Y}}(\mu _1(t),\mu _2(r(t))) \le \epsilon $$.

We show in the proposition below and also in our generalisation results in Sect. 4, that these notions are closely related. However, they also have some fundamental differences, that can be illustrated using the example in Fig. 1.

### Example 2

Consider again the example shown in Fig. 1. We can see that in Fig. 1.(a) $i_{ st }$ and $i_{ dev }$ are trace conformant with value threshold $\epsilon$, as they only exhibit point-wise deviations by values less than $\epsilon$. In contrast, $i_{ st }$ and $i_{ ddev }$ in Fig. 1.(b) are not trace conformant, yet they are hybrid conformant with time and value margins $\tau$ and $\epsilon$, respectively. The key difference is that the inputs depicted in Fig. 1.(b) are very different if compared point-wise, but if one allows for retiming, they are close enough in value after retiming.

The outputs $$o'(i_{ st }) $$ and $$o'(i_{ ddev }) $$ in Fig. 1.(d) illustrate the fundamental difference between hybrid and Skorokhod conformance: although the order of rising and falling signals are reversed in the two trajectories, they are still hybrid conformant, because hybrid conformance disregards the order. However, Skorokhod conformance requires an order-preserving retiming, and hence distinguishes these two trajectories. On the other hand, such retiming exists, e.g., for $$i_{ st }$$ and $$i_{ ddev }$$ in Fig. 1.(b), witnessing their Skorokhod conformance.

We shall use the following notation. We write $\mathsf {Conf}_1 \sqsubseteq \mathsf {Conf}_2$ whenever for all $\mu _1:\mathcal{T}_1 \rightarrow \mathcal{Y}$ and $\mu _2:\mathcal{T}_2 \rightarrow \mathcal{Y}$, we have $\mathsf {Conf}_1(\mu _1,\mu _2)\implies \mathsf {Conf}_2(\mu _1,\mu _2)$. We write $\mathsf {Conf}_1 \sqsubset \mathsf {Conf}_2$ whenever $\mathsf {Conf}_1 \sqsubseteq \mathsf {Conf}_2$ and $\lnot \, \mathsf {Conf}_2 \sqsubseteq \mathsf {Conf}_1$.

### Proposition 1

For any $\tau ,\epsilon \in \mathbb {R}_{\ge 0}$, the following relations hold:

$$ \textsf {TraceConf}_{\epsilon }\sqsubset \textsf {SkorConf}_{\tau ,\epsilon }\sqsubset \textsf {HybridConf}_{\tau ,\epsilon } $$

*Robust Cleanness.* We shall now state the original definition of robust cleanness from [15], adapted to our framework of hybrid systems. It is based on Definition 7 and Proposition 19 from [15]; the phrasing below abstracts from the so-called parameters of interest and standard inputs. Moreover it is cast in the setting of generalised timed traces rather than discrete-step programs, and stated using trace conformance with different thresholds for inputs and outputs, $\kappa _I$ and $\kappa _O$.

Intuitively, a hybrid system is robustly clean if for every pair of input prefixes on which no difference in the inputs exceeding $\kappa _I$ has occurred so far (i.e., all sub-prefixes are trace conformant), the corresponding sets of output prefixes are also conformant with respect to $\kappa _O$. As we consider nondeterministic systems, Hausdorff distance is used to compare sets of outputs (see [15] for details).

### Definition 4

A hybrid system *H* is robustly clean, denoted $$\textsf {RobustClean}$$$$(\kappa _I,\kappa _O)$$, whenever:

$$\begin{array}{l} \forall i_1, i_2 \in GTT (\mathcal{Y}): \forall t\in dom (i_1) \cup dom (i_2): \\ \big (\forall t' \le t:\, \textsf {TraceConf}_{\kappa _I}(i_1[\dots t'], i_2[\dots t']) \,\implies \,\\ \quad \ \big ((\forall o_1\in H (i_1) \, \exists o_2 \in H (i_2):\,\, \textsf {TraceConf}_{\kappa _O}(o_1[\dots t], o_2[\dots t]))\;\wedge \\ \quad \ \ (\forall o_2\in H (i_2) \, \exists o_1 \in H (i_1):\,\, \textsf {TraceConf}_{\kappa _O}(o_1[\dots t], o_2[\dots t]))\big ) \end{array} $$

Note that in the above definition we do not require that $dom(i_1) = dom (i_2)$. In practice, robust cleanness is typically applied to pairs of traces that are both defined over $\mathbb N$. Here, however, for the sake of generality we impose no such restriction. In particular, when the time domains of two traces are different, for example disjoint, the predicate $\textsf {RobustClean}$ will trivially evaluate to $true$.

### Example 3

Consider the traces depicted in Fig. 1. The input prefixes $$i_{ st }$$ and $$i_{ ddev }$$ are given in Fig. 1.(b), and the corresponding pair of outputs is shown in Fig. 1.(c). The trace $$i_{ st }$$ results in output $$o(i_{ st }) $$ and $$i_{ ddev }$$ results in $$o(i_{ ddev }) $$. Suppose that $$\epsilon < |i_{ st }(t_0) - i_{ ddev }(t_0)|$$ and $$\epsilon < |o(i_{ st }) (t_1) - o(i_{ ddev }) (t_1)|$$ at some time $$t_1$$. Thus, the left-hand side of the implication in the Definition 4 instantiated with $$\kappa _I = \kappa _O = \epsilon $$ does not hold for any $$t'$$. Hence, regardless of the outputs, this pair of inputs satisfies the condition of $$\textsf {RobustClean}(\epsilon ,\epsilon )$$, and, if these are the only traces in a hybrid system *H* then we can conclude that *H* is $$\textsf {RobustClean}(\epsilon ,\epsilon )$$.

## Conformance-Based Cleanness

We now define a general notion of conformance-based cleanness and provide two instantiations based on the conformance notions defined in the previous section. The need for considering disturbance in time as well as in value is motivated by our running example from Fig. 1. One of the challenges in performing doping tests for cyber-physical systems is that in such systems timing is rarely perfectly precise, due to imprecision in measurements, or caused by the interaction with the physical world. As illustrated in Example 1, for instance, when checking for software doping in a car [10], the input to the system is the value of the car’s speed over time, which is under the control of a driver, and can thus vary from one execution to the other, even if the driver is trying to execute the same input sequence. Clearly, those variations can be in value, as well as in time.

### Example 4

Consider the test setup sketched in Fig. 1. There, $$i_{ st }$$ and $$i_{ ddev }$$, depicted in Fig. 1.(b) define speed of a car as a function of time. These two input sequences follow a trajectory of values differing by a small margin $$\epsilon $$ (the difference in value allowed by the standard defining the doping tests), but also shifted by a small unit of time $$\tau $$. Observe further that $$|i_{ st }(t_0) - i_{ ddev }(t_0)| \gg \epsilon $$. Thus, without allowing for deviations in time when comparing these input sequences, they will be considered sufficiently different, and as a result their respective exhaust emission outputs will fall out of the comparison when checking for doping according to Definition 4, even if the $$\mathrm {NO}_x$$ emission values in the corresponding outputs $$ H (i_{ st }(t))$$ and $$ H (i_{ ddev }(t))$$ are vastly different, as depicted in Fig. 1.(c). This results in a false negative, i.e., failing to detect a clearly doped system.

In the above example, we demonstrated that not accounting for timing disturbances when relating input trajectories can result in false negatives in doping detection. Dually, using the traditional comparison for output traces can result in false positives by requiring overly strict matching of outputs.

The above example motivates the need to account for timing deviations in trajectories. Intuitively, for input trajectories this relaxation results in considering more traces as conforming, and thus enforcing more comparisons when checking if a system is clean. For output trajectories this means relaxing the conformance requirement by considering two output sequences as conforming even if their values are not perfectly aligned in time. Furthermore, different types of timing deviations need to be considered in different scenarios, for example, depending on whether the order in which values occur is important or not.

### Example 5

Consider the testing workflow from Example 1 and Fig. 1, where inputs $$i_{ st }$$ and $$i_{ ddev }$$ are passed to a car. In the second experiment, depicted in Fig. 1.(d), the car outputs $$o'(i_{ st }) $$ and $$o'(i_{ ddev }) $$, which are hybrid conformant for $$\epsilon $$ and $$\tau $$. Hence this observation of the system is classified as clean under hybrid output conformance. However, the output $$o'(i_{ ddev }) $$ is clearly suspicious, as the values in $$o'(i_{ ddev }) $$ and $$o'(i_{ st }) $$ are reversed. This motivates considering conformance notions that require retimings to be order-preserving. Indeed, using Skorokhod conformance we can detect that the system is doped.

The above examples show that in order to be useful in a diverse set of applications, a software cleanness theory should allow for using a variety of conformance notions. To this end, we next take a more general view on conformance notions, in order to be able to develop a generic conformance-based cleanness framework.

So far, we have defined three specific notions of conformance which either coincide, or are closely inspired by ones that have appeared in the literature. In order to define a general framework for cleanness, we also wish to treat notions of conformance in a more generic manner. To this end, we propose an abstract definition of conformance predicates. As conformance predicates admit variations in time, as well as in value, our definition is based on *retimings*, a device that will play a key role in the context of this work. In its general form a retiming is a pair of functions between two time domains. Intuitively, given two GTTs, a retiming will define a mapping from points in each of the traces to points in the other trace. Note that in general the mappings are not required to be injective; this way we can cater for notions of conformance allowing for the so-called local disorder phenomenon (in particular hybrid conformance – see Proposition 2).

### Definition 5

A *retiming* is a pair of functions between two time domains, i.e., a pair of the form $(r_1,r_2)$, where $r_1: \mathcal{T}_1 \rightarrow \mathcal{T}_2$ and $r_2: \mathcal{T}_2 \rightarrow \mathcal{T}_1$, with time domains $\mathcal{T}_1,\mathcal{T}_2 \subseteq \mathbb {R}_{\ge 0}$. Given two time domains $\mathcal{T}_1$ and $\mathcal{T}_2$, we denote the set of all retimings between $\mathcal{T}_1$ and $\mathcal{T}_2$ with $\mathcal{R}\mathcal{E}\mathcal{T}(\mathcal{T}_1,\mathcal{T}_2)$.

Retiming is explicitly present in the definition of Skorokhod conformance; there, each Skorokhod retiming is required to be a strictly increasing continuous bijection. We can express a Skorokhod retiming *r* as an instance of our definition as the pair $(r,r^{-1})$. In fact, one can also define hybrid conformance, as well as a whole class of conformance notions, using a suitable *family* of retimings.

A family of retimings $\mathsf {Ret}$ can be further constrained by $\tau$ to a subset $\mathsf {Ret}_\tau$ of $\mathsf {Ret}$ containing only functions that shift time by at most $\tau$ time units. In order to use a family of retimings for concrete sequences $\mu _1$ and $\mu _2$, it is necessary to consider only functions that match the domains of the sequences. This leads to a generic notion of conformance associated with a given family of retimings $\mathsf {Ret}$, a given time threshold $\tau$ and a given value threshold $\epsilon$.

### Definition 6

Let $\mathsf {Ret}$ be a family of retimings, and let

$$ \begin{array}{lll} \mathsf {Ret}_{\tau } &{}\!{\mathop {=}\limits ^{\triangle }}\!&{} \{(r_1,r_2) \in \mathsf {Ret}\,\,|\,\, \forall t \in dom (r_i):\, \,|r_i(t)-t| \le \tau \,\,\,(i=1,2) \}, \\ \mathsf {Ret}_{\tau }(\mathcal{T}_1,\mathcal{T}_2) &{}\!{\mathop {=}\limits ^{\triangle }}\!&{} \mathsf {Ret}_{\tau } \cap \mathcal{R}\mathcal{E}\mathcal{T}(\mathcal{T}_1,\mathcal{T}_2). \end{array} $$

A *conformance notion* with time threshold $\tau$ and value threshold $\epsilon$ induced by $\mathsf {Ret}$ is a predicate $\mathsf {Conf}^{\,\mathsf {Ret}}_{\tau ,\epsilon }$ on pairs of GTTs such that, for $\mu _1:\mathcal{T}_1 \rightarrow \mathcal{Y}$, $\mu _2:\mathcal{T}_2 \rightarrow \mathcal{Y}$:

$$ \begin{array}{rl} \mathsf {Conf}^{\,\mathsf {Ret}}_{\tau ,\epsilon }(\mu _1,\mu _2) \iff \exists (r_1,r_2) \in \mathsf {Ret}_{\tau }(\mathcal{T}_1,\mathcal{T}_2): &{} \forall t \in \mathcal{T}_1:\, d_{\mathcal{Y}}(\mu _1(t),\mu _2 \circ r_1(t)) \le \epsilon \\ \,\wedge \, &{} \forall t \in \mathcal{T}_2:\, d_{\mathcal{Y}}(\mu _2(t),\mu _1 \circ r_2(t)) \le \epsilon . \end{array} $$

Using the above definition, we can easily express the specific notions of conformance defined in the previous section by selecting a suitable family of retimings.

### Proposition 2

The conformance predicates below coincide with the notions of conformance induced by the corresponding families of retimings:

- $\textsf {TraceConf}_{\epsilon }$ is induced by the family of retimings containing only identity functions: $\mathsf {Ret}_{\mathsf {id}} = \{(\mathsf {id},\mathsf {id})\mid \mathsf {id} : \mathcal{T}\rightarrow \mathcal{T}\text {is the identity on some }\mathcal{T}\subseteq \mathbb {R}_{\ge 0}\}.$
- $\textsf {SkorConf}_{\tau ,\epsilon }$ is induced by the family of retimings $\mathsf {Ret}= \{(r,r^{-1}) \mid r \text { is a strictly increasing continuous bijection} \}.$
- $\textsf {HybridConf}_{\tau ,\epsilon }$ is induced by pairs of arbitrary functions.

Definition 6 also enables us to define other notions of conformance, such as, for instance a “shift conformance", which, intuitively, shifts all time points by a given constant $c \in \mathbb {R}$, i.e., $\mathsf {Ret}_c = \{(r,r^{-1}) \mid \, r(t)=t+c \}.$

Next, we define a generic notion of cleanness, parametrised by conformance predicates for the input and for the output traces. Instantiating these predicates with existing or new conformance notions, yields different conformance-based notions of cleanness that can capture a variety of cleanness specifications.

We now extend the notion of robust cleanness [15] to allow for “small” variations in time, in addition to the variations in value. To this end, the new notion makes use of two conformance predicates, one that postulates when two input traces should be considered close enough, and another one that specifies when two output traces are close enough.

Our starting point, the notion of robust cleanness in Definition 4, is based on comparison of matching prefixes of a pair of input traces and the corresponding prefixes of the associated output traces. As we now want to accommodate for distance in time, we (1) compare prefixes using a conformance relation, and (2) allow for variation in the length of the compared prefixes that is within the corresponding time-distance threshold. More precisely, when comparing two prefixes, we allow for discarding start and end segments of length at most $\tau$.

This intuition is formalized by the predicate $\mathsf {PrefConf}$ for relaxed comparison of GTT prefixes using a notion of conformance $\mathsf {Conf}$ with tolerance threshold $\tau$ for time disturbance. We use cascaded notation to define $\mathsf {PrefConf}$ as a higher-order function taking $\mathsf {Conf}$ as its first argument. The predicate $\mathsf {PrefConf}$ compares two prefixes $\mu _1$ and $\mu _2$ by requiring that there exist traces $\mu _1[t_1^s\dots t_1^e]$ and $\mu _2[t_2^s\dots t_2^e]$ obtained from them, that are conformant with respect to $\mathsf {Conf}$. These traces are obtained by possibly removing a sub-prefix of length at most $\tau$, and/or removing extending with a suffix of length at most $\tau$.

### Definition 7

Let $\mathsf {Conf}$ be a notion of conformance on GTTs with tolerance threshold $\tau$ for time disturbance. For any pair of GTTs $\mu _1:\mathcal{T}_1 \rightarrow \mathcal{Y}$, $\mu _2:\mathcal{T}_2 \rightarrow \mathcal{Y}$, and $t \in \mathcal{T}=\mathcal{T}_1 \cup \mathcal{T}_2$, the predicate $\mathsf {PrefConf}$ is defined as:

$$ \begin{array}{lll} \mathsf {PrefConf}(\mu _1,\mu _2,t) &{}\!\!\!\!\!\ \iff \ \!\!\!\!\!\!&{} \exists t_1^s \in \! [0,\tau ] \cap \mathcal{T}_1, \exists t_1^e \in \! [t-\tau ,t+\tau ] \cap \mathcal{T}_1,\\ &{} &{} \exists t_2^s \in \! [0,\tau ] \cap \mathcal{T}_2, \exists t_2^e \in \! [t-\tau ,t+\tau ] \cap \mathcal{T}_2\!\!: \\ &{} &{} \mathsf {Conf}(\mu _1[t_1^s\dots t_1^e], \mu _2[t_2^s\dots t_2^e]). \end{array} $$

The predicate $\mathsf {PrefConf}$ provides a generic notion of prefix-conformance. By instantiating it with conformance relations $\mathsf {Conf}_ I$ and $\mathsf {Conf}_ O$ for input and output traces respectively, we define the notion of $(\mathsf {Conf}_ I ,\mathsf {Conf}_ O )$-cleanness.

For deterministic systems $(\mathsf {Conf}_ I ,\mathsf {Conf}_ O )$-cleanness requires that for all pairs of input prefixes for which all sub-prefixes are prefix-conformant w.r.t. $\mathsf {Conf}_ I$, the corresponding pair of output prefixes are prefix-conformant w.r.t. $\mathsf {Conf}_ O$.

### Definition 8

A deterministic system $H$ is $(\mathsf {Conf}_ I ,\mathsf {Conf}_ O )$-clean if

$$\begin{array}{lll} \forall i_1, i_2 \in &{} GTT (\mathcal{Y}) :\, \forall t\in dom (i_1) \cup dom (i_2): &{} \\ &{} (\forall t' \le t:\, \mathsf {PrefConf}_ I (i_1, i_2,t')\big ) \,\implies \, \mathsf {PrefConf}_ O ( H (i_1), H (i_2),t). &{} \end{array} $$

The above definition naturally generalises to nondeterministic hybrid systems, by comparing sets of possible output prefixes using Hausdorff distance as in [15].

### Definition 9

A system $H$ is $(\mathsf {Conf}_ I ,\mathsf {Conf}_ O )$-clean if

$$\begin{array}{l} \forall i_1, i_2 \in GTT (\mathcal{Y}): \forall t\in dom (i_1) \cup dom (i_2): \\ \big (\forall t' \le t:\, \mathsf {PrefConf}_ I (i_1, i_2,t')\big ) \,\implies \,\\ \qquad \ \big ((\forall o_1\in H (i_1) \, \exists o_2 \in H (i_2):\,\, \mathsf {PrefConf}_ O (o_1, o_2,t ))\;\wedge \\ \qquad \ \ (\forall o_2\in H (i_2) \, \exists o_1 \in H (i_1):\,\, \mathsf {PrefConf}_ O (o_1, o_2,t ))\big ). \end{array} $$

Robust cleanness [15] can be now formulated as conformance-based cleanness, which establishes that $(\mathsf {Conf}_ I ,\mathsf {Conf}_ O )$-cleanness is a generalisation. Using hybrid conformance, we define hybrid-conformance cleanness, and similarly, plugging in Skorokhod conformance, we define Skorokhod-conformance cleanness. Formally:

- A hybrid system *H* is robustly clean, denoted $\textsf {RobustClean}(\kappa _I,\kappa _O)$, if and only if *H* is $(\textsf {TraceConf}_{\kappa _I},\textsf {TraceConf}_{\kappa _O})$-clean.
- A hybrid system *H* is *hybrid-conformance clean with conformance thresholds* $(\tau _I,\epsilon _I,$ $\tau _O,\epsilon _O)$, which we denote by $\textsf {HybridClean}(\tau _I,\epsilon _I,\tau _O,\epsilon _O)$, if and only if *H* is $(\textsf {HybridConf}_{\tau _I,\epsilon _I},$ $\textsf {HybridConf}_{\tau _O,\epsilon _O})$-clean.
- A hybrid system *H* is *Skorokhod-conformance clean with conformance thresholds* $(\tau _I,\epsilon _I,\tau _O,\epsilon _O)$, denoted $\textsf {SkorClean}(\tau _I,\epsilon _I,\tau _O,\epsilon _O)$, if and only if *H* is $(\textsf {SkorConf}_{\tau _I,\epsilon _I},$ $\textsf {SkorConf}_{\tau _O,\epsilon _O})$-clean.

We will now establish some key relations between the cleanness notions defined previously. We begin by lifting the implication between conformance relations to implication between cleanness notions defined using those relations.

### Proposition 3

Suppose that $\mathsf {Conf}^{\,1}_I \sqsupseteq \mathsf {Conf}^{\,2}_I$ and $\mathsf {Conf}^{\,1}_O \sqsubseteq \mathsf {Conf}^{\,2}_O$. Then for any system *H*: *H* is $(\mathsf {Conf}^{\,1}_I,\mathsf {Conf}^{\,1}_O)$-clean $\,\implies \,$ *H* is $(\mathsf {Conf}^{\,2}_I,\mathsf {Conf}^{\,2}_O)$-clean.

The proposition above has two important corollaries. The first one explains the relationships between the original robust cleanness, and notions of cleanness based on Skorokhod conformance and hybrid conformance, in particular stating the conservative generalisation property for the latter notions. The second corollary compares cleanness notions with different conformance thresholds.

### Corollary 1

For all $\tau _I,\tau _O,\epsilon _I,\epsilon _O \in \mathbb R_{\ge 0}$, the following implications hold:

1. $$\textsf {RobustClean}(\epsilon _I,\epsilon _O) \implies $$ $$\textsf {SkorClean}(0,\epsilon _I,\tau _O,\epsilon _O) \implies $$ $$\textsf {HybridClean}(0,\epsilon _I,$$$$\tau _O,\epsilon _O)$$,
2. $$\textsf {HybridClean}(\tau _I,\epsilon _I,0,\epsilon _O)\,\implies \,$$ $$\textsf {SkorClean}(\tau _I,\epsilon _I,0,\epsilon _O)\,\implies \,$$ $$\textsf {RobustClean}$$$$(\epsilon _I,\epsilon _O)$$.

Also, $\textsf {RobustClean}(\epsilon _I,\epsilon _O)=\textsf {SkorClean}(0,\epsilon _I,0,\epsilon _O)= \textsf {HybridClean}(0,\epsilon _I,0,\epsilon _O)$ and hence $\textsf {SkorClean}$ and $\textsf {HybridClean}$ are conservative extensions of robust cleanness.

### Corollary 2

For all $\epsilon _ I ,\epsilon '_ I , \epsilon _ O ,\epsilon '_ O ,\tau _ I ,\tau '_ I , \tau _ O ,\tau '_ O$ that satisfy the inequalities

$\epsilon '_ I \le \epsilon _ I ,\quad \tau '_ I \le \tau _ I ,\quad \epsilon '_ O \ge \epsilon _ O ,\quad \tau '_ O \ge \tau _ O$ the following implications hold:

1. $$\textsf {RobustClean}(\epsilon _ I ,\epsilon _ O ) \,\implies \,$$ $$\textsf {RobustClean}(\epsilon '_ I ,\epsilon '_ O )$$;
2. $$\textsf {HybridClean}(\epsilon _ I ,\tau _ I ,\epsilon _ O ,\tau _ O ) \,\implies \,$$ $$\textsf {HybridClean}(\epsilon '_ I ,\tau '_ I ,\epsilon '_ O ,\tau '_ O )$$;
3. $$\textsf {SkorClean}(\epsilon _ I ,\tau _ I ,\epsilon _ O ,\tau _ O ) \,\implies \,$$ $$\textsf {SkorClean}(\epsilon '_ I ,\tau '_ I ,\epsilon '_ O ,\tau '_ O )$$.

### Example 6

Consider the testing workflow in Fig. 1. The inputs passed to a car are $$i_{ st }$$ and $$i_{ ddev }$$, depicted in Fig. 1.(b). One of the test results is presented in Fig. 1.(c), where $$i_{ st }$$ reveals output $$o(i_{ st }) $$ and $$i_{ ddev }$$ reveals $$o(i_{ ddev }) $$. We assume that $$\epsilon < |i_{ st }(t_0) - i_{ ddev }(t_0)|$$ and $$\epsilon < |o(i_{ st }) (t_1) - o(i_{ ddev }) (t_1)|$$ at some time $$t_1$$.

- For inputs $i_{ st }$ and $i_{ ddev }$, any output is immediately deemed $\textsf {RobustClean}(\epsilon ,\epsilon )$, as the left-hand side of the implication in Definition 8 does not hold for any $t'$. Note, that for other inputs the car used for testing might not be $\textsf {RobustClean}(\epsilon ,\epsilon )$.
- As explained in Example 2, $$i_{ st }$$ and $$i_{ ddev }$$ are hybrid conformant for $$\epsilon $$ and $$\tau $$, i.e., the predicate $$\mathsf {PrefConf}_I$$ on the left-hand side of the implication in Definition 8 holds. $$\mathsf {PrefConf}_O$$, however, fails at time $$t_1$$ for signals $$o(i_{ st }) $$ and $$o(i_{ ddev }) $$. Hence, the system tested in Fig. 1.(c) is not $$\textsf {HybridClean}(\epsilon ,\tau ,\epsilon ,\tau )$$.

We now discuss testing and falsification of conformance-based cleanness.

For systems with discrete time domains the existing methods for verifying [15] or testing [10] robust cleanness can be readily applied.

In the case of hybrid cleanness, existing methods for testing hybrid conformance, such as [2] and [4] can be extended to testing and falsification of hybrid cleanness of hybrid systems consisting of traces with finite time domains. Methods for checking Skorokhod conformance were presented in [17]. Due to the quantification over all time-points $t'$ in our Definition 8 and Definition 9, it is not clear how to directly extend them to testing Skorokhod cleanness.

## Case Study

In this section we evaluate the proposed notion of hybrid cleanness in the context of doping detection in relation to the recent Diesel Emissions Scandal.

Conducting software doping tests for cyber-physical systems has a range of applications. A prominent example is the body of recent work [8–10, 14, 15, 32, 34] that gives insights into the Diesel Emissions Scandal. This is a world-wide scandal where millions of diesel cars have been equipped with defeat devices reducing the effectiveness of emission cleaning systems during real-world usage – in contrast to the regulator-defined driving scenarios on a chassis dynamometer, where the amount of emitted pollutants are well below the applicable limits.

Assuming the existence of a contract that formalizes when software is considered to be doped, recent work demonstrates how doping tests can be generated automatically and how the characteristic challenges arising with these kinds of tests can be tackled [10]. A major challenge is the distortion of inputs that can occur during test execution. As doping tests have to be conducted on the final product, i.e., a vehicle such as a passenger car, a human driver has to provide the inputs to the car by driving it. It is far from trivial to provide the inputs exactly as defined by the test case. Official regulations, that define the approval process for new car models, precisely specify test cycles for which they allow tolerances in the input of up to 2 km/h (in car speed). But even driving a car within this tolerance requires a very experienced driver. To strengthen the position of consumers against manufacturers, it is necessary to allow manufacturer-independent methods to check the compliance of a car model with the applicable regulations, i.e., the absence of defeat devices. These methods are supposed to require a reasonable amount of effort, and training a driver over months so that she has enough experience to stay within the tolerance of 2 km/h is way beyond reasonable effort. This means that the responsibility for accounting for the driver’s imprecision must be shifted to the techniques for checking for software doping.

In this section we give a short summary of recent doping tests with a diesel car and demonstrate how the theory developed in this paper addresses the above challenge. More precisely, it allows us to overcome the imprecise timing leading to minor input distortions, by appropriately accounting for the effect of retiming on the input value error. We further show how using our theory one of the tests reveals strong indications for a defeat device in the car under test – despite a very inexperienced driver conducting the test. This doping detection would not have been possible using the cleanness notions existing prior to this work.

**Fig. 2.**
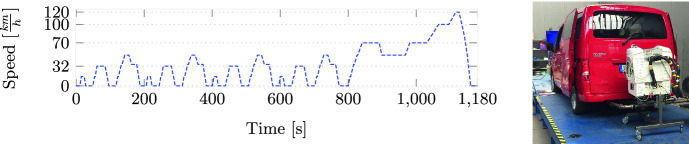
Left: New European Driving Cycle (NEDC); Right: test setup with Nissan NV200 Evalia on a chassis dynamometer attached to a PEMS.

*Physical Set-Up of the Experiment.* Before a car model can be sold, it must meet the requirements defined in the official regulations. The type approval procedure requires the car to be placed on a chassis dynamometer. Cars have to follow certain standardized test cycles, each defined as a function from time to speed. One of the test cycles, involved in the diesel scandal, was the New European Driving Cycle (NEDC) [42] shown in Fig. 2. For the tests here, we consider the *speed* of the car as *input*, since this is the parameter defining a test cycle. The *total amount of* $\mathrm {NO}$ *and* $\mathrm {NO}_2$ *(abbreviated as* $\mathrm {NO}_x$) is the only *output* of interest.

The car under test is a Nissan NV200 Evalia, with Renault 1.5 dci (110hp) diesel engine and approved w.r.t. regulation *Euro 6b*. The test set-up is shown in Fig. 2.

In order to perform a check for defeat devices using a cleanness test, we consider, in addition to the original NEDC, two manually synthesized tests. These test cycles, denoted PowerNEDC and SineNEDC were proposed in previous work [10] and are defined as follows. PowerNEDC is based on the NEDC but slightly deviates from it by enforcing higher accelerations ($1.5\frac{\text {m}}{\text {s}^2}$ instead of $0.94\frac{\text {m}}{\text {s}^2}$) after 56 s, 251 s, 446 s and 641 s. The *maximum input deviation from* NEDC *is* $\kappa _I$ = 10 km/h. SineNEDC is defined as the NEDC superimposed by a sine curve, formally ${{{SineNEDC}}}(t) = \max \{0, {{{NEDC}}}(t) + 5\sin (0.5t) \}$, with a *maximum input deviation from* NEDC *of* $\kappa _I$ = 5 km/h.

**Fig. 3.**
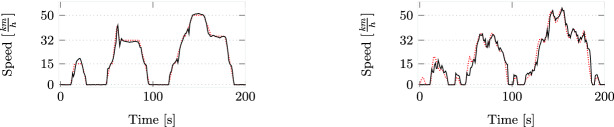
Initial 200s of PowerNEDC (left) and SineNEDC (right) planned test cycles (red, dotted) and actually driven (black). (Color figure online)

These test cycles are defined by specifying the input value (the car’s speed) in each second. Both test cases are shown by the red dotted lines in Fig. 3.

*Conformance-Based Cleanness Tests for* NEDC*.* We have applied our theory of conformance-based cleanness to check for doping, i.e., the presence of a defeat device, in the car under test. For this, we have at our disposal the raw data obtained from three test drives: (1) Test drive  is the result of NEDC cycle driven by a human driver. It serves as the reference behaviour of the car, to which we will compare the executions of the other two test cycles. (2) Test drive  is the trajectory that is produced as the result of a human driver driving PowerNEDC. (3) Test drive  is the trajectory that is produced as the result of a human driver driving SineNEDC.

The values of the actual sequences of inputs executed by driving the car are sampled in steps of 0.05 s. As mentioned earlier, the human in the loop makes testing considerably more challenging. The maximum deviation of inputs compared to the test specification for NEDC is just below 10 km/h, for PowerNEDC is almost 12 km/h, and for SineNEDC it approaches 16 km/h. This shows that the perturbation introduced by the human driver is clearly noticeable. The amount of $\mathrm {NO}_x$ measured for  is 180 mg/km, for  the measurements revealed 204 mg/km and 584 mg/km, respectively.

In order to detect doping (by falsifying cleanness), the input sequences of  have to be each compared to  , and if the input sequences in the corresponding pair are conforming, then the respective outputs (the total $\mathrm {NO}_x$ emission values) have to be checked for conformance.

As we desire for our doping tests to be as strict as possible, we identify hybrid conformance $\textsf {HybridConf}_{\tau _ I ,\epsilon _ I }$, i.e., the weakest of the conformance relations discussed in Sect. 3, as the most suitable conformance relation for the comparison of input traces. As the outputs are just single values, the choice of output conformance relation is immaterial in this case, so we take $\textsf {HybridConf}_{0,\epsilon _ O }$.

Formally, we consider the deterministic hybrid system $H$ defined by the input GTTs  , and check whether $H$ is $\textsf {HybridClean}(\tau _I,\epsilon _I,0,\epsilon _O)$-clean for given values of $\tau _I$, $\epsilon _I$ and $\epsilon _O$.

The driver’s imprecision has a significant effect on the values in the input sequences and their timing. This can lead to dismissing pairs of sequences if they are incorrectly deemed too far apart, and thus missing some indications of doping. For instance, a too strict comparison of  to  will dismiss this pair of executions; however, the measured $\mathrm {NO}_x$ emission during the  drive is *three times more* than the one measured during  .

Testing $\textsf {HybridClean}(\tau _I,\epsilon _I,0,\epsilon _O)$ allows us to perform a realistic comparison by taking into account the two possible sources of driving errors: the over- or undershooting of the speed, and the timing offsets, where the driver accelerates or decelerates too fast or too slowly. In comparison, prior doping tests based on Robust Cleanness, considered only the former, i.e., the point-wise offset in speed. As we demonstrate, depending on the specified value threshold, there are cases when this is insufficient to identify doping. Indeed, looking into the official regulations, we can see that they allow for a timing variation of one second [19, 42]. Thus, essentially, the regulations allow for hybrid conformance with $\tau _ I = 1$ s.

**Hybrid Cleanness Testing.** In order to test $\textsf {HybridClean}(\tau _I,\epsilon _I,0,\epsilon _O)$ we have to examine the conformance relations  and  between the corresponding input sequences. Recall that since the output of the system measured in each test is the total amount of $\mathrm {NO}_x$ emitted during the test, i.e., a single value for the whole execution, timing plays no role when quantifying the value error for the output.

In order to evaluate the power of using hybrid cleanness for detecting doping, we consider different values for $\epsilon _ I$ and $\tau _ I$, and perform two types of analysis of the results of testing $\textsf {HybridClean}(\tau _I,\epsilon _I,0,\epsilon _O)$, which we describe below.

**Effect of** $\tau _ I$ **on the Minimal** $\epsilon _ I$ **for Which Inputs are Conforming.** First, we fix a maximum value that we allow for the time offset $\tau _ I$. For this $\tau _ I$ we analyse our dataset to find the minimal $\epsilon _ I$ such that for the combination $\tau _ I$ and $\epsilon _ I$ the input traces under consideration satisfy hybrid conformance. For $\tau _ I = 0$ we get exactly the $\epsilon _ I$ for which the two traces are trace conformant. Table 1 (left side) shows the computed $\epsilon _ I$ values for $\tau _ I = 0, 0.5, 1, 2, 5,10$.

As expected, when we increase $\tau _ I$, the minimal $\epsilon _ I$ decreases. At some point (at $\tau _ I = 2$ for PowerNEDC and $\tau _ I = 5$ for SineNEDC) the decrease in the value error reduces notably. This happens because the error is only partially caused by the incorrect timing of the driver.

From the values reported in Table 1 (left) we see that if, for example, we allow deviation for the input $\tau _ I =1$, as per the official regulation, and set $\epsilon _ I =15$, then we have that both  and  are true, while, for $\tau _ I =0$ both are false. Thus, under hybrid conformance these pairs of traces will be considered in the cleanness test, while under trace conformance they will be dismissed.

**Table 1.** Value thresholds for fixed $\tau _ I$ (left) and time thresholds for fixed $\epsilon _ I$ (right). Values are given as mg/km and time in seconds.

	$\tau _ I = 0$	$\tau _ I = 0.5$	$\tau _ I = 1$	$\tau _ I = 2$	$\tau _ I = 5$	$\tau _ I = 10$	$\epsilon _ I = \kappa _I$	$\epsilon _ I = \kappa _I\!+\!2$
Power	$\epsilon _ I = 15.88$	$\epsilon _ I = 15.03$	$\epsilon _ I = 12.41$	$\epsilon _ I = 10.10$	$\epsilon _ I = 10.07$	$\epsilon _ I = 10.07$	$\tau _ I = 67.35$	$\tau _ I = 10.8$
Sine	$\epsilon _ I = 16.17$	$\epsilon _ I = 15.46$	$\epsilon _ I = 14.24$	$\epsilon _ I = 12.91$	$\epsilon _ I = 11.67$	$\epsilon _ I = 11.37$	$\tau _ I = 72.4$	$\tau _ I = 4.05$

Since the difference between the outputs measured during  and during  is vast, we establish that $\textsf {HybridClean}(1,15,0,180)$ does *not* hold.

**Effect of** $\epsilon _ I$ **on the Minimal** $\tau _ I$ **for Which Inputs are Conforming.** Second, we fix the maximum value error $\epsilon _ I$ and examine what minimal $\tau _ I$ results in a combination $\tau _ I$ and $\epsilon _ I$ for which the analysed data is hybrid conformant. For the synthesized test cases we study the error tolerance $\epsilon _ I$ set to the respective input thresholds $\kappa _I$. As discussed above, this is 10 km/h for PowerNEDC and 5 km/h for SineNEDC. We also consider the scenario where the error tolerance allowed by the official regulation for the test cycle is added, that is, we also consider $\epsilon _ I = \kappa _I$ + 2 km/h. The two rightmost columns of Table 1 show the necessary time shifts to achieve these value errors. As apparent, they reduce by approximately 84% and 94% when adding the error tolerance of 2 km/h.

These values for $\tau _ I$ give us the minimal tolerance threshold for time, for which $\textsf {HybridClean}(\tau _ I ,\epsilon _ I ,0,180)$ is violated in $H$ for the given $\epsilon _ I$; the value of $\epsilon _O$ is fixed at 180 mg/km according to the standard [10].

*Evaluation and Discussion.* The analysis of the data shows that it is indeed necessary to not only consider a deviation of value, but to also allow for timing deviations, especially when the quality of the studied driving tests suffers from the human-caused input distortions. In terms of the theory established in this paper, this means that in scenarios like this one, employing $\textsf {HybridClean}$ is more adequate than using prior notions such as $\textsf {RobustClean}$, and without this, the cases of doping we have detected would go unnoticed. Allowing a retiming of up to 10.8 s (for PowerNEDC) and of 4.05 s (for SineNEDC) makes both inputs conformant to the NEDC input, so we are able to detect the violation of SineNEDC for the hybrid cleanness for the specified desired value error tolerance. While these time deviations appear large given the test cycle timeline, they are acceptable when we recall that the tests are executed by human drivers.

If, on the other hand, we want to restrict the tolerance in time to one second, we are able to consider both tests for the hybrid cleanness for value error tolerance of 12.41 km/h for PowerNEDC and 14.24 km/h for SineNEDC.

This demonstrates how conformance-based cleanness notions like $\textsf {HybridClean}$ allow us to some extent to account for human-caused errors related to timing.

Finally, while hybrid cleanness is arguably the appropriate notion for the case study considered here, our generic theory of conformance-based cleanness allows for using other conformance notions as appropriate for the CPS under test.

## Conclusions

In this paper, we presented a theory of doping detection and cleanness based on the notions of conformance for cyber-physical systems. Our new notion accounts for possible “deviations” of the system output, upon “perturbing” its inputs, both in time and in values. Both notions of “deviation” and “perturbation” turn out to be expressible using a generic notion of retiming. We instantiate our definition with specific notions of retiming from the conformance testing literature. We apply our notions to a case study from the automotive domain and demonstrate how our generalised notions are useful in using actual driving cycles for doping detection according to the New European Driving Cycle (NEDC) [42].

We intend to turn our theory into an automatic tool for doping detection, using hybrid systems models. We intend to use the HyConf tool [4] as the starting point and use our search-based testing implementation in HyConf [5] to automate the process of test-case generation and test-case selection. Once this process is automated, one can generate test-cases that can go beyond a specific standard and detect intelligent defeat devices that cheat the standards and the tests prescribed by them.

We also intend to organise widespread experiments regarding emission detection to put our theory into practice. Our experimental set-up involves instrumenting a large number of cars using low-cost equipments, constructing models of emission behaviour, and generating realistic driving scenarios that are more likely to detect doping.
